# Supplementary material for: DMRforPairs: identifying Differentially Methylated Regions between unique samples using array based methylation profiles
Source: BMC Bioinformatics. 2014 May 15;15:141. doi: 10.1186/1471-2105-15-141 (PMC4046028; doi:10.1186/1471-2105-15-141)
Supplement: Additional file 2 — DMRforPairs output for the comparison of A431-MCF7 and NA17018-NA17105. Please start from the HTML files in each folder. Available via the BMC Bioinformatics website. [file 1471-2105-15-141-S2.zip › 1394847754114233_MOESM2_ESM/A431_MCF7/figures/18034.pdf]

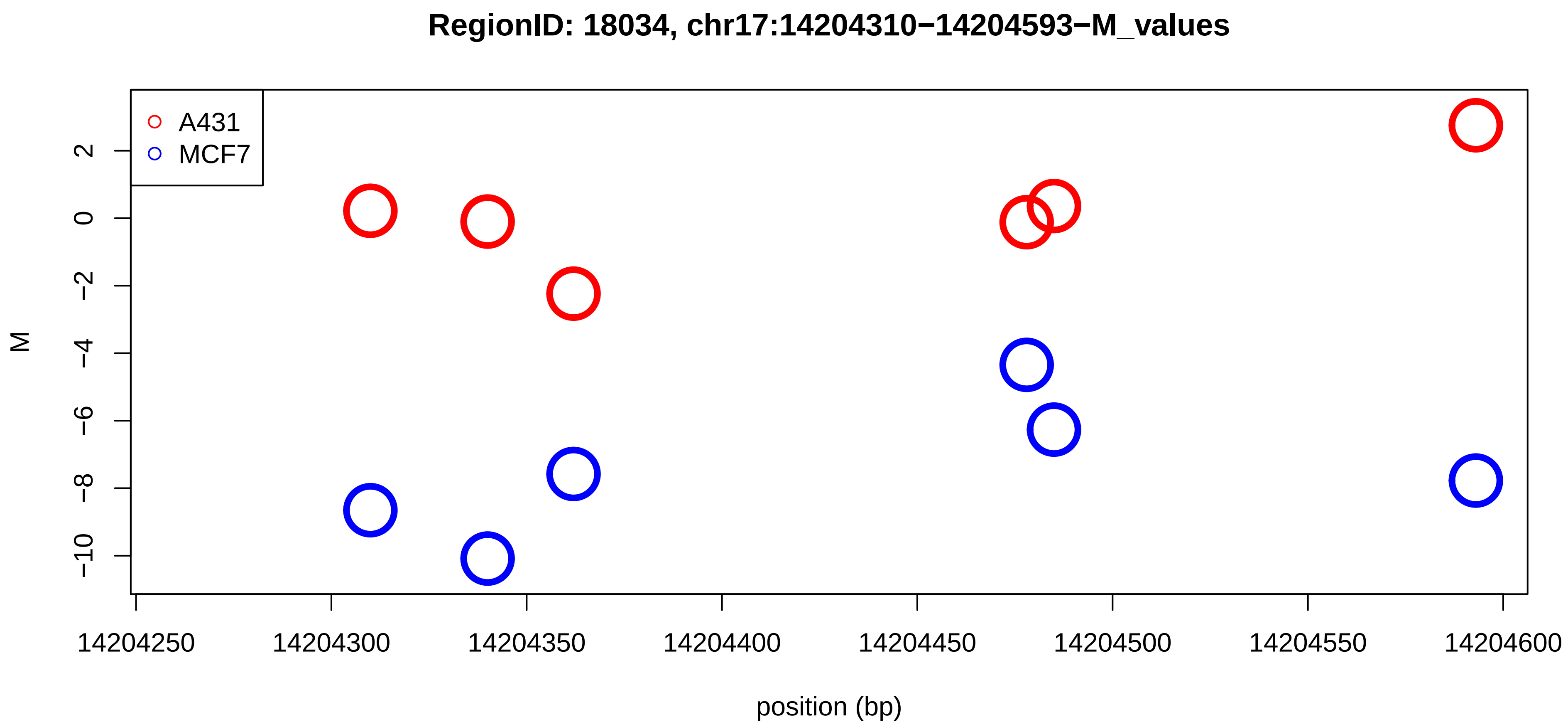
RegionID: 18034, chr17:14204310−14204593−M_values
A431
MCF7
M
2
0
−2
−4
−6
−8
−10
14204250
14204300
14204350
14204400
14204450
14204500
14204550
14204600
position (bp)

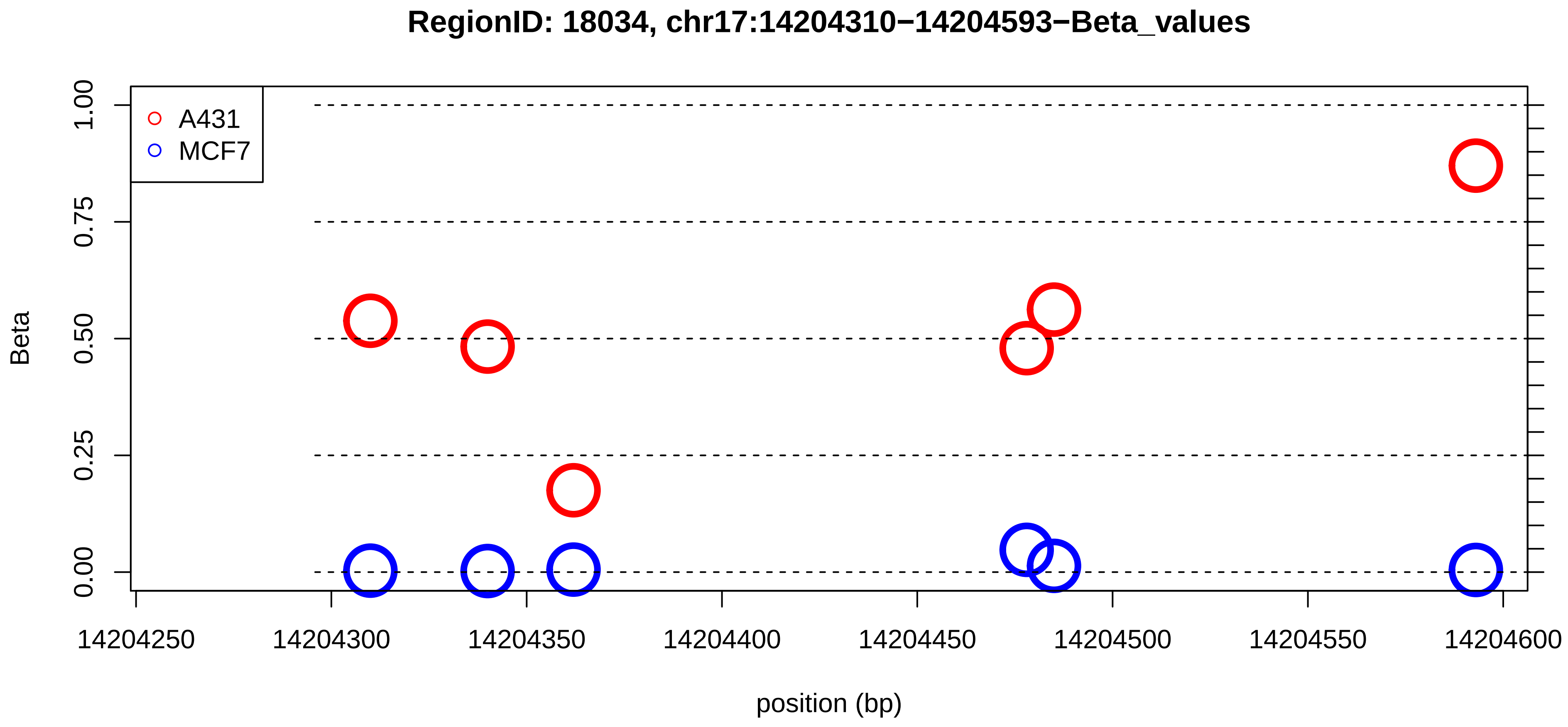
RegionID: 18034, chr17:14204310−14204593−Beta_values
A431
MCF7
Beta
1.00
0.75
0.50
0.25
0.00
14204250
14204300
14204350
14204400
14204450
14204500
14204550
14204600
position (bp)

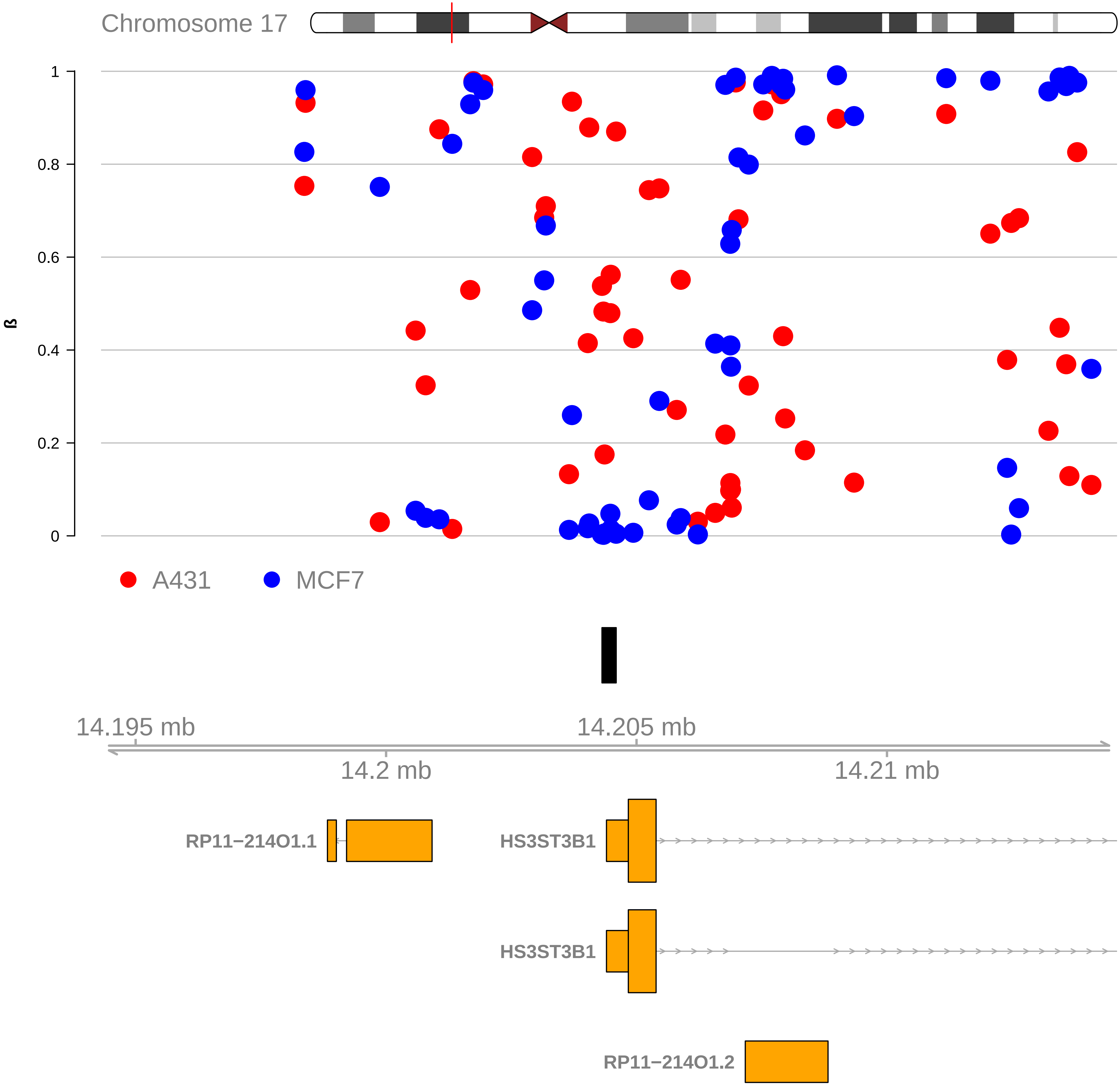
Chromosome 17
1
0.8
0.6
0.4
0.2
0
ß
A431
MCF7
14.195 mb
14.205 mb
14.2 mb
14.21 mb
RP11−214O1.1
HS3ST3B1
HS3ST3B1
RP11−214O1.2
